# Supplementary material for: A Novel Betulinic Acid Analogue: Synthesis, Solubility, Antitumor Activity and Pharmacokinetic Study in Rats
Source: Molecules. 2023 Jul 28;28(15):5715. doi: 10.3390/molecules28155715 (PMC10419975; doi:10.3390/molecules28155715)
Supplement: Supplementary file 1 [file molecules-28-05715-s001.zip › molecules-2473720-supplementary.pdf]

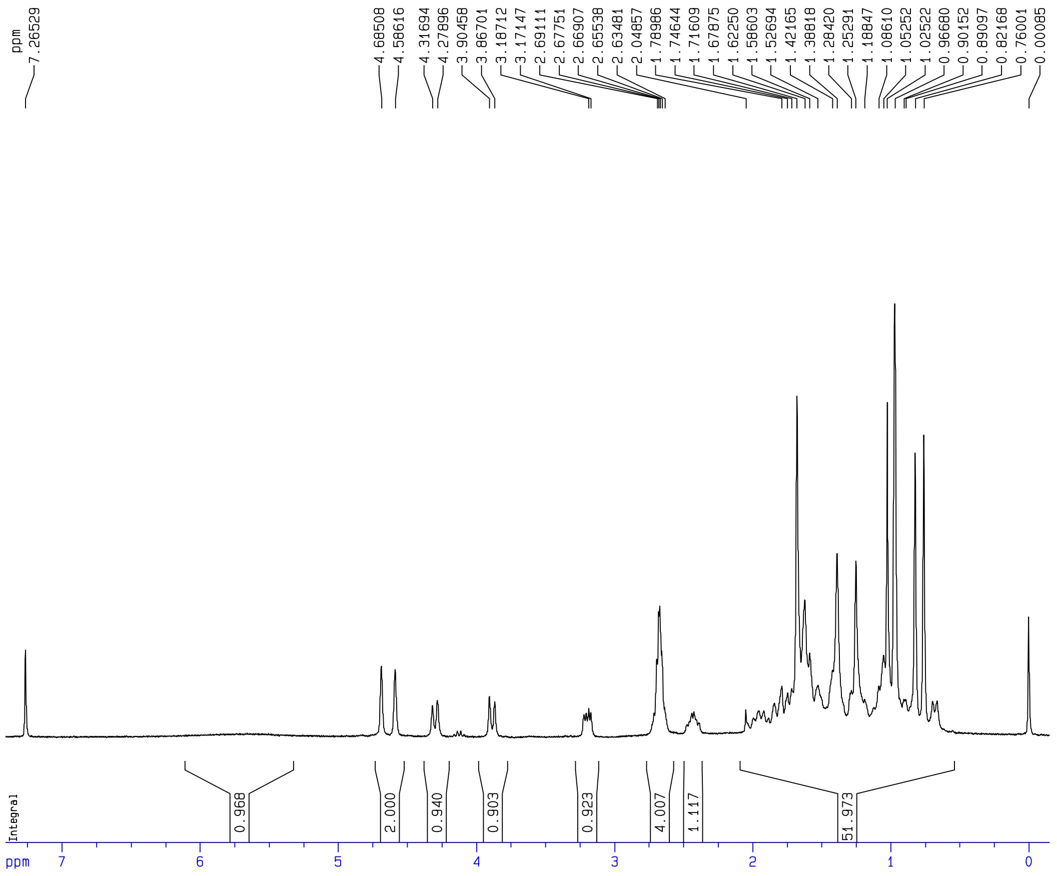

Supplementary Figure S1. ^{1}H NMR spectra of SBE

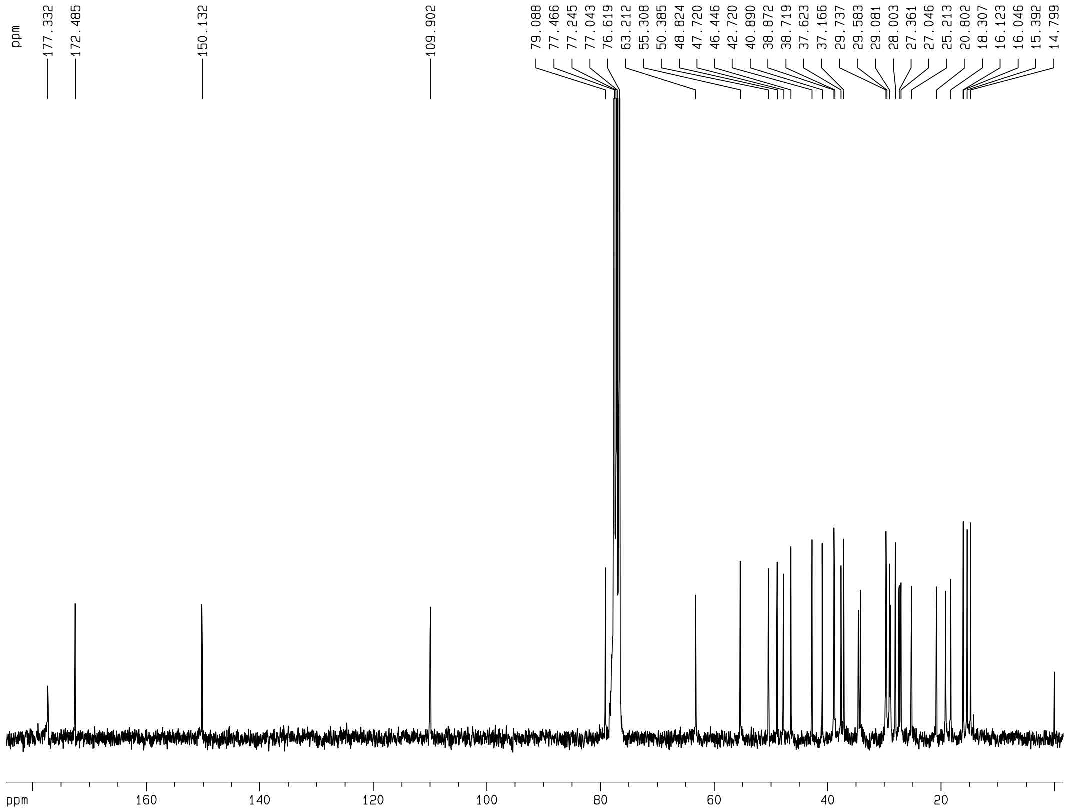

Supplementary Figure S2. ^{13}C NMR spectra of SBE

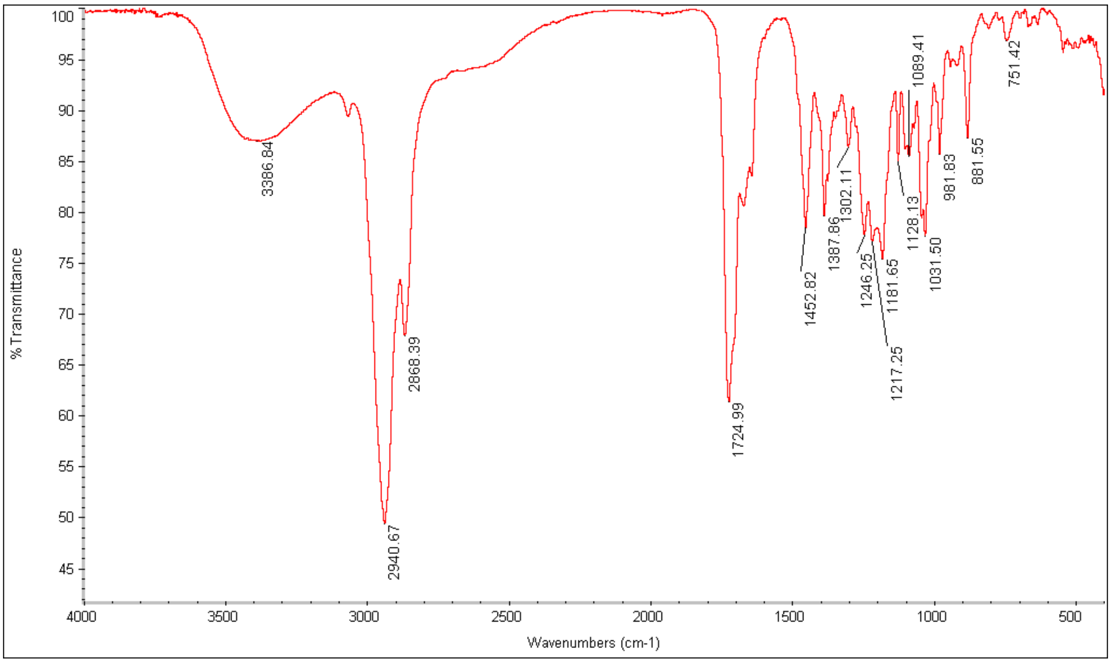

Supplementary Figure S3. IR spectra of SBE

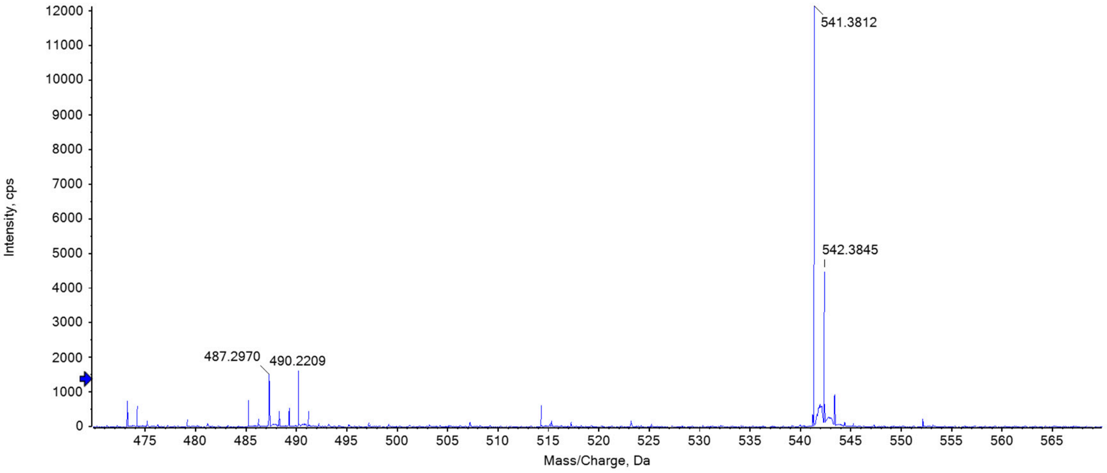

Supplementary Figure S4. HRMS spectra of SBE

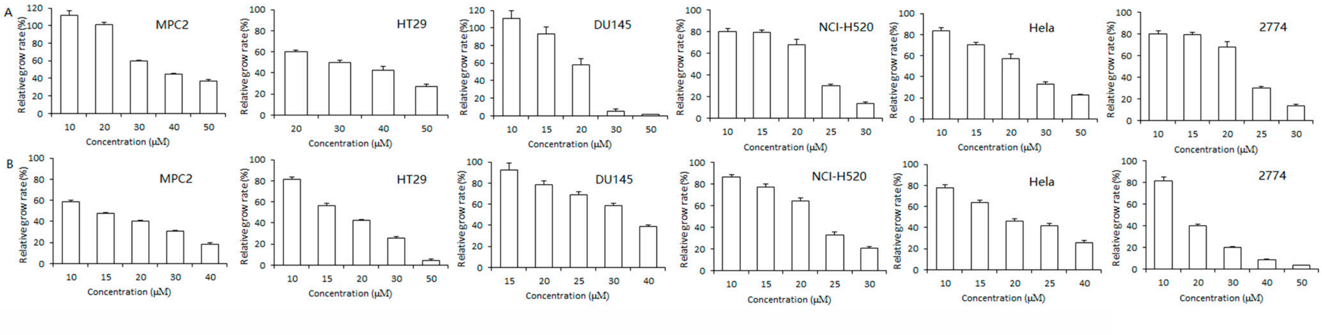

Supplementary Figure S5. Relative growth rate of different cancer cells incubated with different concentration of BA (A) and SBE (B).

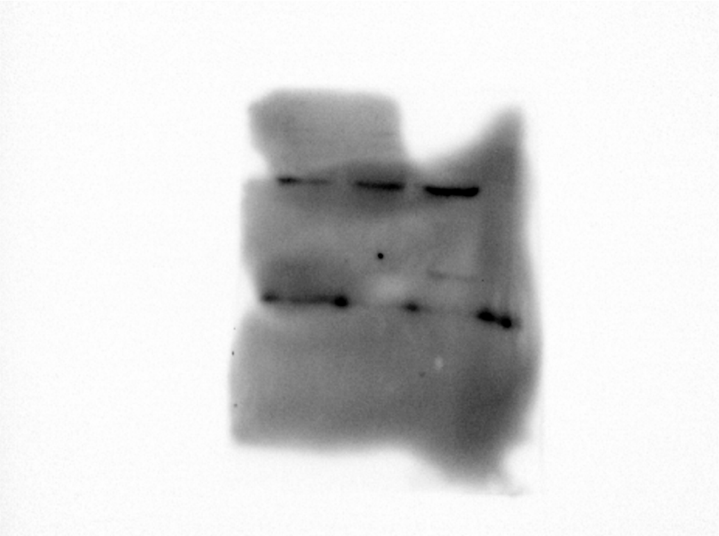

Supplementary Figure S6. Expression level of Bad (The upper ones).

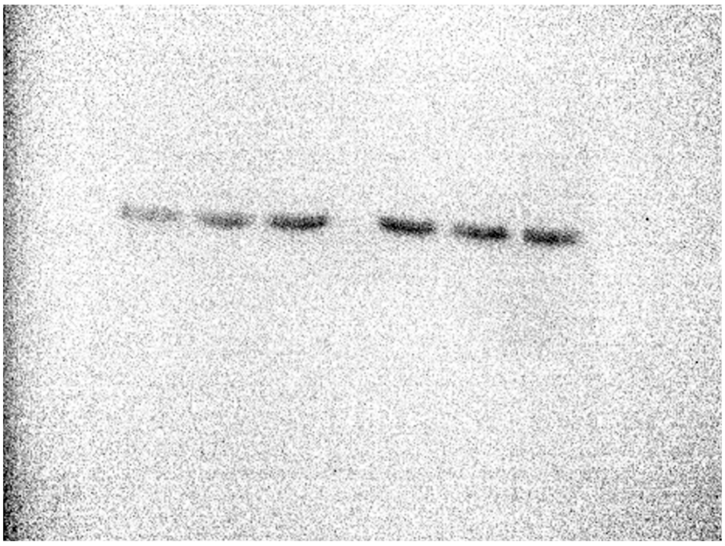

Supplementary Figure S7. Expression level of Bcl-xL.

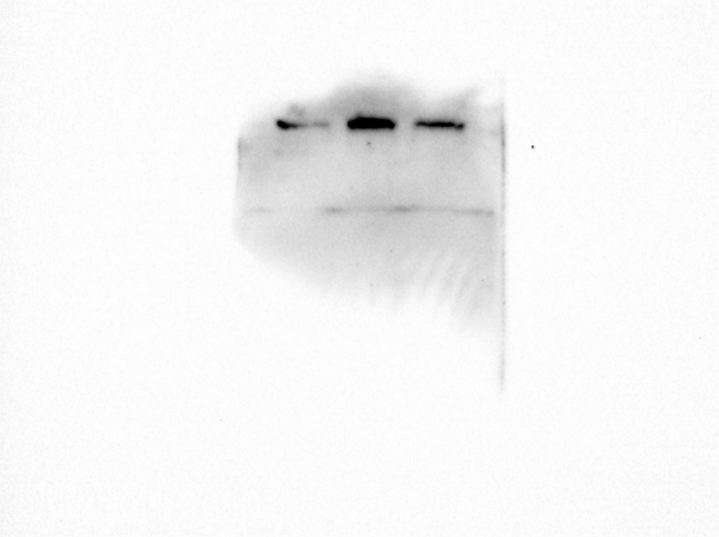

Supplementary Figure S8. Expression level of Cleaved-Cas9.
